# Analyzing the methanation thermodynamic feasibility of steel plant byproduct gases

**DOI:** 10.1038/s41598-024-62982-4

**Published:** 2024-05-29

**Authors:** Qiang Ling, Xue Li, Qin Pei, Zhao Lei, Ping Cui, Rui Lun Xie

**Affiliations:** 1 Anhui Key Laboratory of Coal Clean Conversion and Utilization, School of Chemistry and Chemical Engineering, Anhui University of Technology, Ma’anshan City, 243002 China; 2 School of Finance and Economics, Wanjiang University of Technology, Ma’anshan City, 243002 China

**Keywords:** Methanation, Byproduct gases, Thermodynamic equilibrium, Aspen plus, Energy science and technology, Mathematics and computing

## Abstract

To improve the utilization of byproduct gases in the steel plant, the coke oven gas (COG) methanation combined with blast furnace gas (BFG) and basic oxygen furnace gas (BOFG) was proposed in viewpoint of economy and environment. The optimization mathematics model based on Gibbs free energy minimization was established to predict the thermodynamic feasibility of the proposed methanation. To solve the proposed model, the convenient method was implemented by using the Gibbs module in Aspen Plus software. Effects of operation parameters on the methanation performance were revealed to identify the optimized conditions. To reduce the solid carbon concentration, it was found that the optimized conditions of temperature, pressure and stoichiometric number were 650 °C, 30 bar and 3.0, respectively. Moreover, it was discovered that 10 mol% of BFG or BOFG could be mixed into COG to obtain the maximum methane yield. In addition, it was testified that there were the good agreements between calculated results and industrial and published data, which indicated that the proposed methanation was thermodynamically feasible. Therefore, the simple and easy method was developed to evaluate the methanation operating conditions from the aspect of thermodynamic equilibrium, which provided the basic process conditions of byproduct gases methanation to enhance the steel plant efficiency and reduce carbon emissions.

## Introduction

Natural gas was regarded as the eco-friendly fossil energy, and widely used in industry and transportation due to the high heating value (HHV, 37.26–28.10 MJ/m^3^)^1^. Unfortunately, the current energy distribution in China is rich in coal, lacking in gas, and short in petroleum^2,3^. Thus, the synthetic natural gas (SNG) was developed by using the abundant resource of coal^4^. The SNG based on coal was realized by using coal gasification, which had the characteristics of high energy consumption and high carbon emission^5^. Furtherly, the H_2_/CO ratio of syngas need to be adjusted to the range from 3 to 3.5, which was suitable to the methane production. The water–gas shift reaction (CO + H_2_O → CO_2_ + H_2_) was implemented to adjust the H_2_/CO ratio, which resulted in the high carbon emission and the additional costs. Therefore, the alternative methane synthesis route was greatly drawn attention by using byproduct gases from other industry.

**Figure 1:**
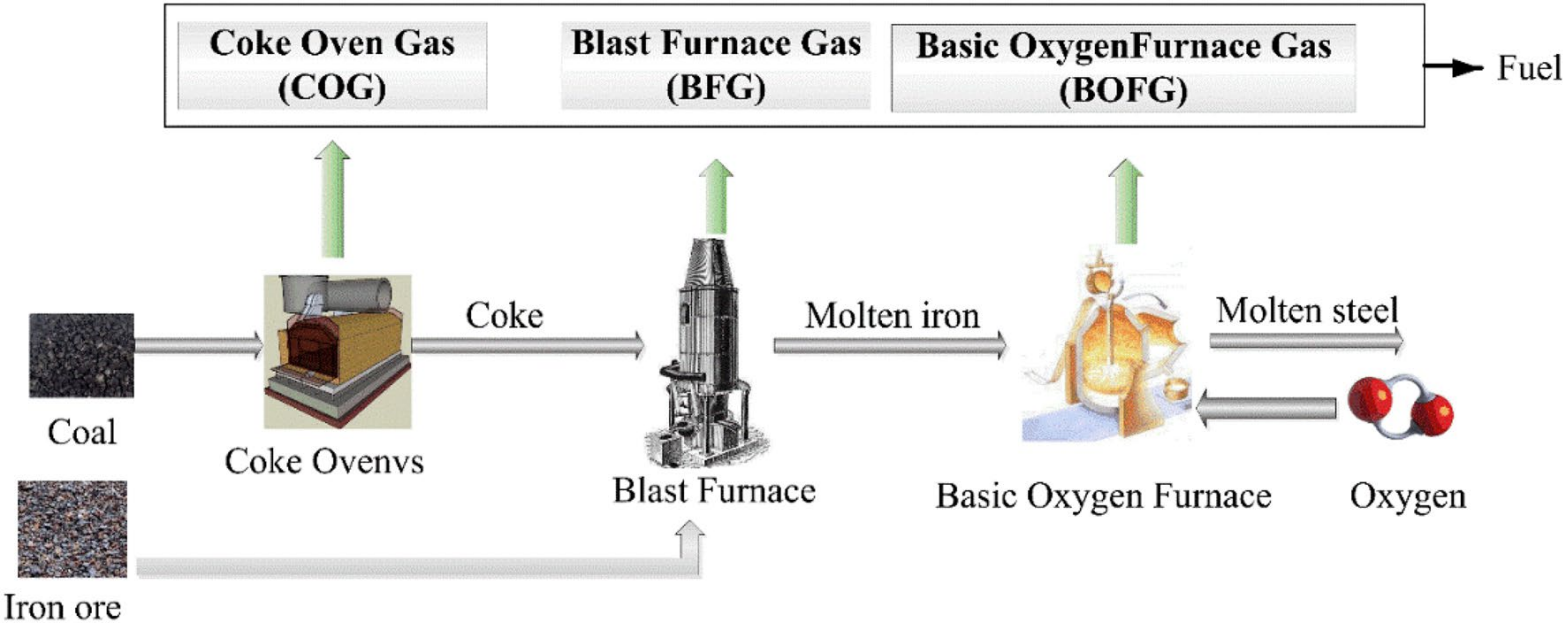
The integrated steelmaking process and the typical utilization of coke gases.

There were three types of byproduct gas in the integrated iron and steel company. As illustrated in Fig. 1, the coke oven gas (COG) was generated in the coke production^6^. Then, the coke and iron ore were fed into a blast furnace, where the blast furnace gas (BFG) was obtained. Afterwards, molten iron and oxygen were fed into a basic oxygen furnace, in which the basic oxygen furnace gas (BOFG) was produced. BFG was often heated by using the double preheating combustion to supply the energy for blast furnace, coke oven and steel rolling, etc. BOFG was mainly used for heating and insulation in the converter workshop, the molten iron pouring and the steel pouring, and so on. COG was mainly used as fuel to heat the coke oven, the steel rolling and the continuous casting, etc. Thus, it was found that byproduct gases in the integrated iron and steel company were primarily burnt to provide the energy, which resulted in the high carbon emission. Therefore, it was necessary to develop the utilization technology of above byproduct gases because the combustion of byproduct gases resulted in the high carbon emission and the wasting of high value substance, such as CO and H_2_. It was pointed out that the optimum ratio (H_2_-CO_2_)/(CO + CO_2_) was 3.0 for methanation^7^. However, the volume fraction of H_2_ and CH_4_ in COG was greater than 80%, which led to a large amount of hydrogen-containing material to be recycled to reactor. This phenomenon resulted in the increasing operating cost and the potential safety hazard. Thus, it was required to adjust the H_2_/CO ratio to be a suitable value. It is found that the effective method for adjusting the H_2_/CO ratio was that COG was mixed with the crude gas from coal gasification, which was used to product different chemicals or fuels, for example methane^8^, methanol^9^, dimethyl ether (DME)^10^, dimethyl carbonate (DMC)^11^. Among above technologies, COG methanation was widely used in industry scale due to the short process and the low investment.

Often, coal gasification was used to supply the carbon source in COG methanation process, which simultaneously led to the high energy consumption and the high carbon emission. In fact, it was found that the volume fraction of CO and CO_2_ in BFG and BOFG was greater than 40% and 70%, respectively. Therefore, BFG and BOFG were used as the carbon source to replace coal gasification, and were combined with COG to produce CH_4_, which was beneficial to the environment and the economy. Furthermore, CO_2_ was in abundance supplied by using the carbon capture use and storage (CCUS), which could provide the required carbon source in COG methanation process. Thus, the COG methanation combined with the CCUS provided the new technology from the viewpoint of environment and economy.

Two kinds of key factors of COG methanation were the catalyst deactivation and the reaction temperature control, respectively. On one hand, the Ni supported catalyst was usually applied in the COG methanation industrial scale. The carbon deposition, the sintering, the poisoning and the instability of catalyst were principal obstacles to effect on the methane production. Furtherly, it was found that the solid carbon that came from side reaction could cause the catalyst carbon deposition. Further, it was discovered that solid carbon was reduced by adjusting the H_2_/CO ratio or adding water into the reaction system^12^. On the other hand, the temperature of methanation reaction was closely related to the catalyst sintering, as well as to the reaction equilibrium and the rate of chemical reaction. It was indicated^13^ that the conversion of CO_2_ and CO were both increased by 1%, which resulted in that the temperature of reactor was raised by 60 and 72 °C, respectively. The rapid increasing temperature was disadvantageous to the methane production from the perspective of the chemical equilibrium because the higher temperature easily contributed to the lower CH_4_ productivity, the more catalyst deactivation, the harder temperature control, and so on. Based on above discussions and our acknowledges, it was clearly pointed out that the chemical equilibrium was strongly associated with the chemical components, the reaction conditions, and the catalyst deactivation. Therefore, the chemical equilibrium of COG methanation combined with BFG and BOFG was implemented in our work to adjust the H_2_/CO ratio and avoid carbon deposition.

The COG methanation is a typical gas–solid multiphase reaction system, including the methanation, water–gas shift, methane cracking, reduction, alcoholization, and so on. The catalyst was consisted by the active center, the support and the promoter, which was interacted each other and influenced on above reactions. To simplify the complex reaction system, the catalyst was ignored in our work since the chemical reaction equilibrium was unchanged with or without catalyst. There were two primary means to estimate thermodynamic equilibrium, which were the equilibrium constants^14^ and the minimization of the Gibbs free energy^15^. The first method was rather complicated and tedious when several reactions simultaneously occur. The second method was implemented by using the process simulation software, such as Aspen Plus, ChemCAD, ProII^16^. Aspen Plus software was the popular process modeling software, and used to deal with complex reaction systems^17^. The thermodynamic equilibrium of CO_2_ reforming reaction to syngas was performed by using process modeling software (Aspen HYSYS) based on the principle of minimization of Gibbs free energy^18^. Thermodynamic equilibrium of the methanation reactions of CO_2_ was investigated with CHEMCAD using the same method^19^. Wang et al.^20^ evaluated the thermodynamic equilibrium of hydrogen production from BFG and COG using HSC Chemistry 7.0 according to the same principle. However, the thermodynamic equilibrium of COG methanation combined with BFG and BOFG was still lacking.

In our work, the thermodynamic equilibriums of COG methanation combined without and with BFG and BOFG were studied by using Aspen Plus software based on the Gibbs free energy minimization. Then, the effects of different factors (temperature, pressure, feeding gas composition) on the methanation performance such as conversion, yield, product selectivity and carbon production were explored. After that, the appropriate feeding gas compositions (or the ratio of COG, BFG and BOFG) for methane production were determined through the optimization, which could provide the technological conditions for COG methanation.

## Materials and methods

### Materials

As shown in Table 1, the volume fractions of COG, BFG and BOFG^21^ were listed, where the mole fraction was calculated using Peng–Robinson equation of state with Boston–Mathias modifications method at temperature (25 °C) and pressure (1 atm). It was clearly found that the H_2_/CO ratio in the COG gas was equal to 0.7571/0.0576, which was equal to 13.14 and much higher than that required ratio of methanation (3.0). Therefore, mixture of COG, BFG and BOFG as feeding gases was a feasible method for methanation by considering their different compositions.

**Table 1:** Composition of the coke gases of steel plant.

Feeding gas	COG		BFG		BOFG	
	Vol fraction	Mole fraction	Vol fraction	Mole fraction	Vol fraction	Mole fraction
H_2_	0.65	0.7571	0.03	0.0392	0.04	0.0516
CO	0.06	0.0576	0.2	0.2153	0.58	0.6160
CH_4_	0.21	0.1317	0	0	0	0
C_2_H_6_	0.03	0.0119	0	0	0	0
CO_2_	0.02	0.0126	0.24	0.1698	0.2	0.1396
N_2_	0.03	0.0290	0.53	0.5756	0.18	0.1928

### Reactions

As presented in Table 1, the main components in gases of three types of furnaces were H_2_, CO, CO_2_, C_2_H_6_, CH_4_, and N_2_, respectively, which were also the reactants during the methanation process. Because the carbon deposition was the important factor for catalyst deactivation, the thermal cracking reactions of hydrocarbons were considered due to the generation of solid carbon in above cracking reaction. It was reported that water–gas shift reaction (R3) and dehydrogenation (R11) also occurred during methanation^3^. Thus, reactions considered in our work were the methanation reaction, the water–gas shift reaction, the disproportionation reaction and the thermal cracking reaction, as outlined in Table 2^3,5,8^.

**Table 2:** Possible reactions involved during methanation with COG, BFG and BOFG.

Number	Reaction	ΔH (kJ/mol)
R1	$CO+3{H}_{2}\leftrightarrow C{H}_{4}+{H}_{2}O$	− 206
R2	$C{O}_{2}+4{H}_{2}\leftrightarrow C{H}_{4}+2{H}_{2}O$	− 165
R3	$CO+{H}_{2}O\leftrightarrow {H}_{2}+C{O}_{2}$	− 31
R4	${CH}_{4}+3{CO}_{2}\leftrightarrow 4CO+2{H}_{2}O$	247
R5	$C+{H}_{2}O\leftrightarrow CO+{H}_{2}$	156
R6	$C+2{H}_{2}O\leftrightarrow C{O}_{2}+2{H}_{2}$	90
R7	$2CO\leftrightarrow C{O}_{2}+C$	− 173
R8	$C+2{H}_{2}\leftrightarrow C{H}_{4}$	− 72
R9	$CO+2{H}_{2}\leftrightarrow C{H}_{3}OH$	− 91
R10	$C{O}_{2}+3{H}_{2}\leftrightarrow C{H}_{3}OH+{H}_{2}O$	− 41
R11	${C}_{2}{H}_{6}\leftrightarrow {C}_{2}{H}_{4}+{H}_{2}$	136
R12	${CH}_{4}\leftrightarrow 2{H}_{2}+C$	76

As shown in Table 2, it was assumed that carbon atoms in CO and CO_2_ were converted into CH_4_, and oxygen atoms were resulted in water. Thus, the stoichiometry for methanation was presented as Eq. (1).

1 $${\text{uCO}}_{{2}} {\text{ + vCO + }}\left( {\text{4u + 3v}} \right){\text{H}}_{{2}} { = }\left( {\text{u + v}} \right){\text{CH}}_{{4}} { + }\left( {\text{2u + v}} \right){\text{H}}_{{2}} {\text{O}}$$

where *u* and *v* denoted the stoichiometric coefficient of CO and CO_2_, respectively.

It was evident that the stoichiometric constraint for methanation was [H_2_] = 3[CO] + 4[CO_2_], where the square bracket denoted the molar number of components. The effect of stoichiometric number (*R*) on methanation was defined, as shown Eq. (2). It was inferred that the stoichiometric number was equal to 3.0 under the ideal reaction condition. When the stoichiometric number was less than 3.0, numbers of solid carbon were increased, which resulted in the catalyst deactivation. When the stoichiometric number was greater than 3.0, a lot of hydrogen was needed to be recycled to reactor, which led to the high operation cost and the poor safety.

2 $$R = \frac{{\left[ {H_{2} } \right] - \left[ {CO_{2} } \right]}}{{\left[ {CO_{2} } \right] + \left[ {CO} \right]}}$$

### Mathematic model

It was well known that the Gibbs free energy was minimum when the equilibrium state of a system was reached. Under the constant temperature and the constant pressure, the Gibbs free energy of a reaction was defined as the difference between products’ and reactants’ Gibbs free energy of formation, as shown in Eq. (3).

3 $${\text{Min}}\left( {\Delta G_{R}^{0} } \right)_{{\text{T, P}}} { = }\left( {\Delta G_{F,Products}^{0} } \right)_{{\text{T, P}}} - \left( {\Delta G_{F,Reactants}^{0} } \right)_{{\text{T, P}}}$$

where ${\left({\Delta G}_{F,Products}^{0}\right)}_{\text{T, P}}$ and ${\left({\Delta G}_{F,Reactants}^{0}\right)}_{\text{T, P}}$ were the Gibbs free energy of formation of products and reactions, respectively. Gibbs free energy of formation was estimated by summing the chemical potentials of all *N* components^22–24^, as described in Eq. (4).

4 $${\text{G}}^{{\text{t}}} { = }\mathop \sum \limits_{{\text{j = 1}}}^{{\text{N}}} {\text{n}}_{{\text{j}}} {\upmu }_{{\text{j}}}$$

where *n*_*j*_ was the number of moles of species *j*, *μ*_*j*_ was the chemical potential of species *j*. The chemical potential of a component was given in Eq. (5).

5 $${\upmu }_{{\text{i}}} { = }\overline{{{\text{G}}_{{\text{i}}}^{{0}} }} { + 8}{\text{.314Tln}}\left( {\frac{{{\text{f}}_{{\text{i}}} }}{{{\text{f}}_{{\text{i}}}^{{0}} }}} \right)$$

where *T* and *f*_i_ were the temperature and the fugacity of species *i*, respectively. The superscript 0 denoted the standard thermodynamic quantity, thus $\overline{{\text{G} }_{\text{i}}^{0}}$ and ${f}_{i}^{0}$ were the standard Gibbs free energy and the standard fugacity of species *i*, respectively. The chemical potential of a component was also presented in terms of pressure^25^ as described in Eq. (6).

6 $${\upmu }_{{\text{i}}} { = }\overline{{{\text{G}}_{{\text{i}}}^{{0}} }} { + 8}{\text{.314}} \times {\text{T}} \times {\text{ln}}\left( {\frac{{\phi {\text{P}}_{{\text{i}}} }}{{{\text{P}}^{{0}} }}} \right)$$

where *ϕ* was the fugacity coefficient, *P*_i_ was the partial pressure of species *i*, *P*_0_ was the standard pressure. Using the Gibbs energy of reaction, the chemical equilibrium constant was also calculated as presented in Eq. (7).

7 $$\Delta G_{R}^{0} = - RTlnK_{e}$$

where T was temperature in Kelvin, R was the universal gas constant, and *K*_e_ was the chemical Equilibrium constant.

### Solution method

**Figure 2:**
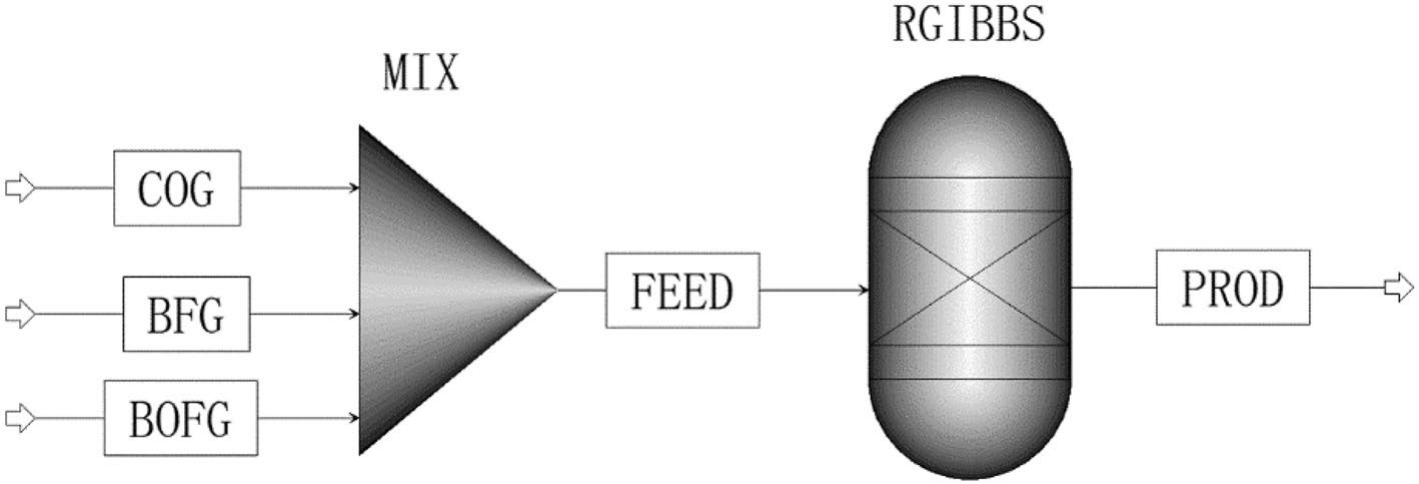
The flowsheet for estimating the thermodynamic equilibrium of methanation.

The mathematic model of Gibbs free energy minimum was consisted by a series of equations from (3) to (7), which was the typical optimization model and was difficult to be solved. To easily solve the Gibbs free energy minimum model, the mathematic model of Gibbs free energy minimum and the optimization mathematic method were integrated into the module, which was the reactor of RGibbs module in Aspen Plus software^26^. Furthermore, the thermodynamic calculation method was crucial for the accuracy of estimation results. It was pointed out that the Peng–Robinson equation of state with Boston-Mathias modifications was suitable for simulating methanation^27^. The methanation flowsheet of Gibbs free energy minimum was developed by using Aspen Plus, as illustrated in Fig. 2.

The flowsheet was consisted by three types of gas stream (COG, BFG and BOFG), two kinds of unit operations (mixer (MIX) and Gibbs reactor (GIBBS)) and one kind of feed out stream (PROD). In details, each section was described as follows:

1. The coke oven gas, the blast furnace gas and the basic oxygen furnace gas were represented by COG, BFG and BOFG, respectively.
2. MIX was a block, where some material streams were mixed into one outlet stream.
3. GIBBS represented the Gibbs Reactor, which was based on the minimization of the Gibbs free energy to calculate the equilibrium^19^, where temperature and pressure in the module need to be specified.

Sensitivity analysis was the useful tool in Aspen Plus, which was used to examine the effects of one or more flowsheet variables on the others^28^. Procedures to perform the sensitivity analysis were given as follows: (1) Specify measured variables, (2) Specify manipulated variables, (3) Specify ranges for manipulated variables, (4) Specify quantity to be computed and tabulate the target parameter. Since the temperature and the pressure were two dominant factors for the methanation process, the ranges of temperature and pressure during implementing the sensitivity analysis were 200–800 °C^29^ and 10–60 bar^30^, respectively. In addition, there were four different scenarios considered in this work, as showed in Table 3.

**Table 3:** Four kinds of feed streams conditions.

Case number	Temperature(°C)	Pressure(bar)	COG	BFG	BOFG
Case 1	200–800	10–60	√		
Case 2	200–800	10–60	√		√
Case 3	200–800	10–60	√	√	
Case 4	200–800	10–60	√	√	√

The first case was defined as the basic scenario, where the COG methanation was only studied. The second case was used to present the COG methanation combined with BOFG methanation. The third case represented the COG methanation combined with BFG methanation. The fourth case stood for the methanation among COG, BOFG and BFG. To evaluate the performance of gases methanation, conversion, selectivity and yield were selected as key parameters, and defined as follows. Moreover, aforementioned parameters were represented by using MATLAB software.

1. Conversions of species *i*,

8 $${\text{C}}_{{\text{m}}} \left( \% \right){ = }\frac{{\left[ {\text{m}} \right]_{{{\text{in}}}} - \left[ {\text{m}} \right]_{{{\text{out}}}} }}{{\left[ {\text{m}} \right]_{{{\text{in}}}} }} \times 1{00}$$

The square bracket stood for the mole flow rate, and *m* was H_2_, CO and CO_2_, respectively.

2. Yields. CH_4_ had two potential resources (CO and CO_2_). Thus, the CH_4_ yield was defined as follows.

9 $${\text{Y}}_{{{\text{CH}}_{{4}} }} { = }\frac{{\left[ {{\text{CH}}_{{4}} } \right]_{{{\text{out}}}} - \left[ {{\text{CH}}_{{4}} } \right]_{{{\text{in}}}} }}{{\left[ {{\text{CO}}} \right]_{{{\text{in}}}} + \left[ {{\text{CO}}_{{2}} } \right]_{{{\text{in}}}} }}$$

3. Selectivity. Since the reactants of methanation were CO and CO_2_ as presented in Table 2, the formula of selectivity was defined as Eq. (10) based on mass balance of carbon atoms.

10 $${\text{S}}\left( \% \right){ = }\frac{{\left[ {{\text{CH}}_{{4}} } \right]_{{{\text{out}}}} - \left[ {{\text{CH}}_{{4}} } \right]_{{{\text{in}}}} }}{{\left[ {{\text{CO}}} \right]_{{{\text{in}}}} - \left[ {{\text{CO}}} \right]_{{{\text{out}}}} + \left[ {{\text{CO}}_{{2}} } \right]_{{{\text{in}}}} - \left[ {{\text{CO}}_{{2}} } \right]_{{{\text{out}}}} }} \times {100}$$

## Results and discussion

### Methanation of COG: case 1

**Figure 3:**
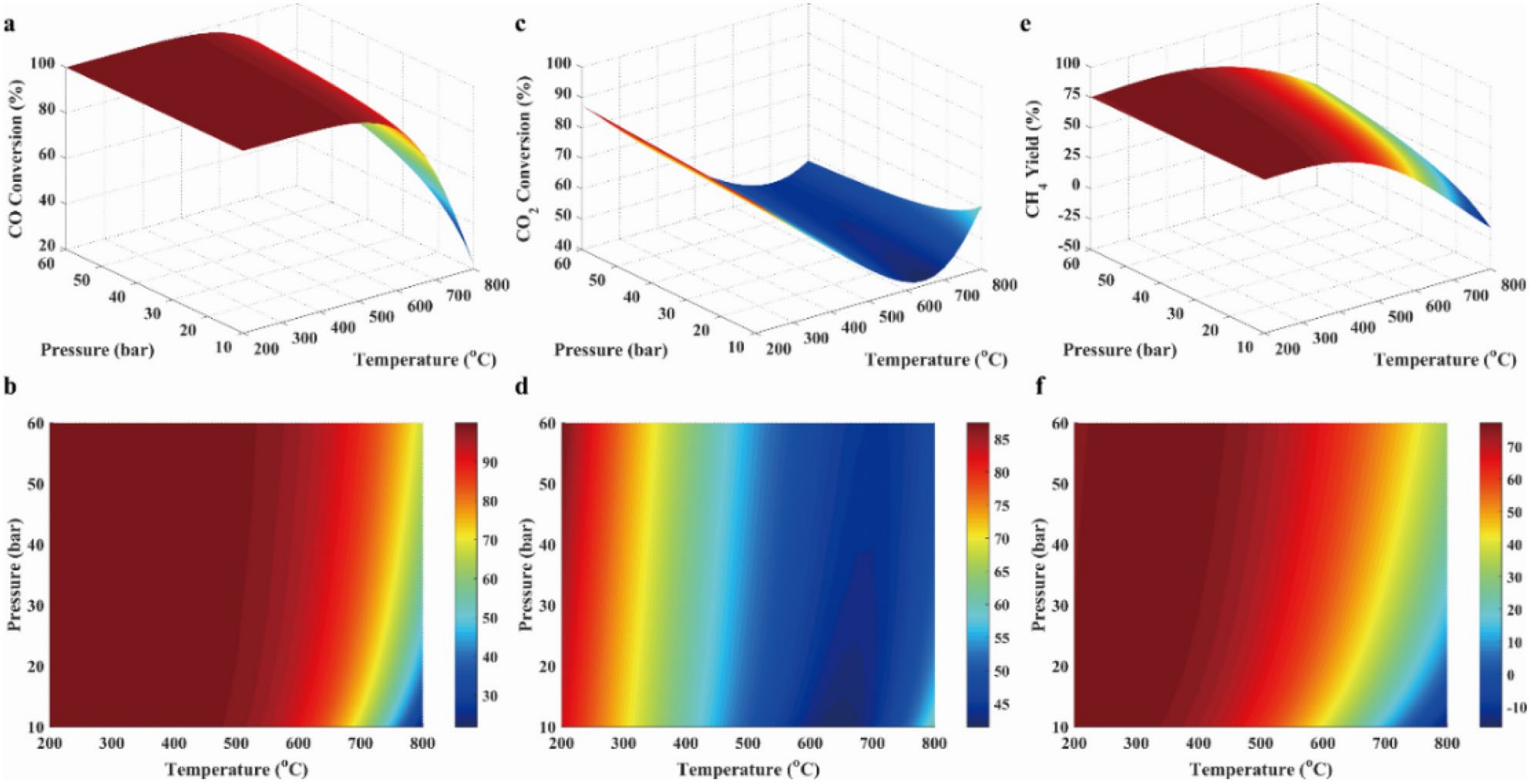
CO conversion (**a**, **b**), CO_2_ conversion (**c**, **d**), and CH_4_ yield (**e**, **f**) in case 1.

The factors that influenced on the synthesizing methane were the pressure, the temperature, the feed component, the feed flow rate, the product component, the product feed rate, the ratio of H_2_/CO, and the stoichiometric number *R*, and so on, which was provided in the Support Materials. In Support Materials, main factors within the large range were studied. It was found that the obvious factors affecting methanation reaction was the temperature, the pressure, the feed ratio and the stoichiometric number *R*. Thus, the influences of temperature, pressure, feed ratio and stoichiometric number on the performances of methanation reaction were shown in the following figures. Conversions of CO and CO_2_, and CH_4_ yield were revealed in Fig. 3, which were shown with 3D surfaces representing the functions of temperature and pressure. As presented in Fig. 3a, the conversion of CO was near to 100% at below 500 °C, and then was decreased with rising temperature when the temperature was above 500 °C. This was because R1 was the exothermic reaction. As displayed in Fig. 3c, the conversion of CO_2_ was decreased with rising temperature at below 650 °C because of the exothermic reaction R2, and was increased with rising temperature at above 650 °C due to the endothermic reaction of R4. As illustrated in Fig. 3e and f, CH_4_ yield was decreased with the increasing temperature because the exothermic reactions (R1, R2 and R8) took place. In addition, it was clearly seen that conversions of CO and CO_2_ were increased with the rising pressure in Fig. 3, which was attributed to the fact that the methanation of CO and CO_2_ were reactions with reduced volume. Furthermore, it was found that the pressure had the insignificant effect on methanation below 650 °C.

**Figure 4:**
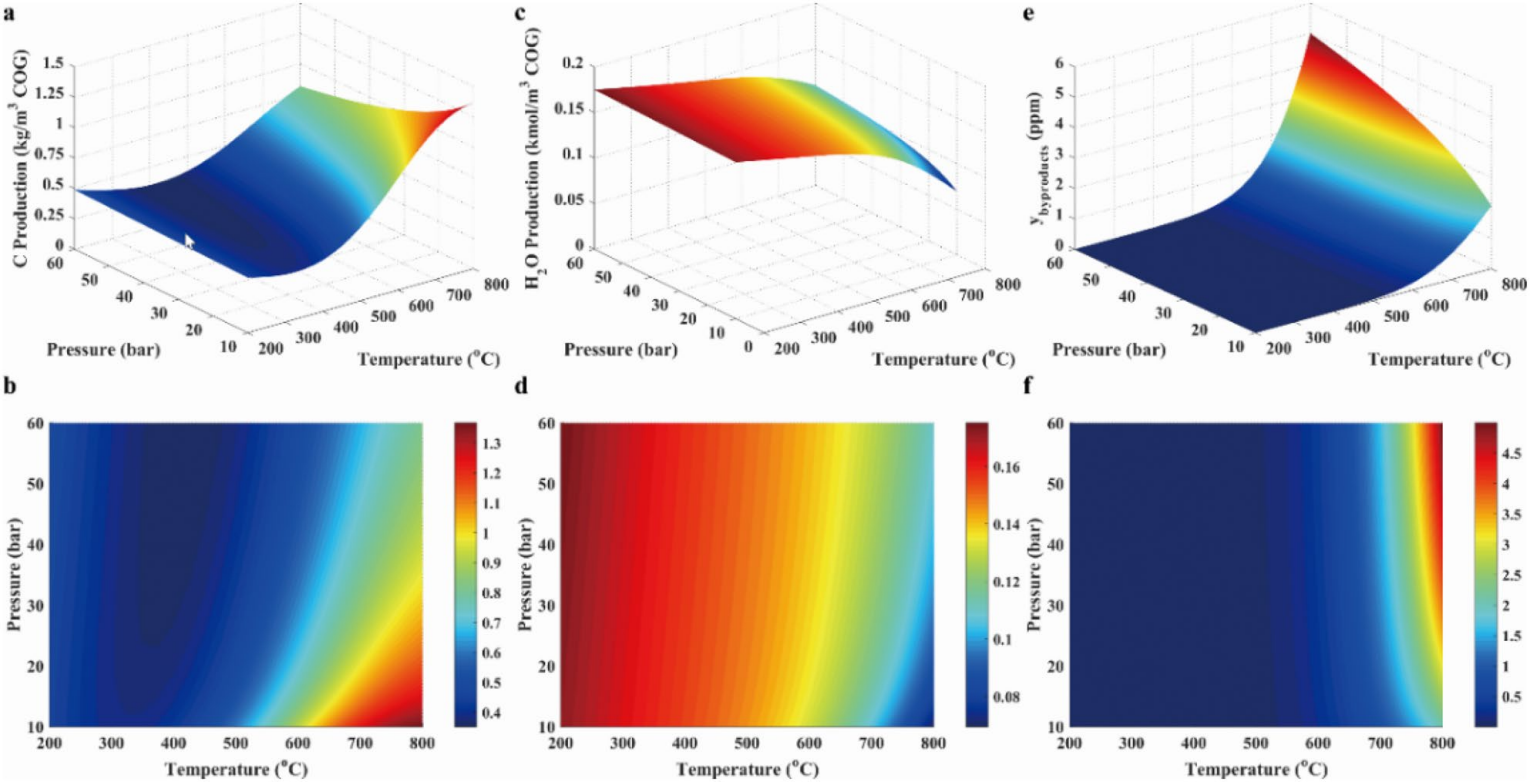
Solid carbon production (**a**, **b**), water (**c**, **d**) and other byproducts (**e**, **f**) in case 1.

As presented in Fig. 4a and b, the solid carbon concentration was increased with the increasing temperature and the decreasing pressure because the increased volume and endothermic reaction (R12) took place. As illustrated in Fig. 4c and d, H_2_O concentration was increased with the decreasing temperature due to exothermic reactions (R1 and R2). Besides, H_2_O concentration was slightly increased with the rising pressure. The reason for the variation was that the iso-volumetric and endothermic reaction reverse water–gas shift (R3 in Table 2) was dominant in the system. As revealed in Fig. 4e and f, other products concentrations were ignored in the system due to the low concentration.

**Figure 5:**
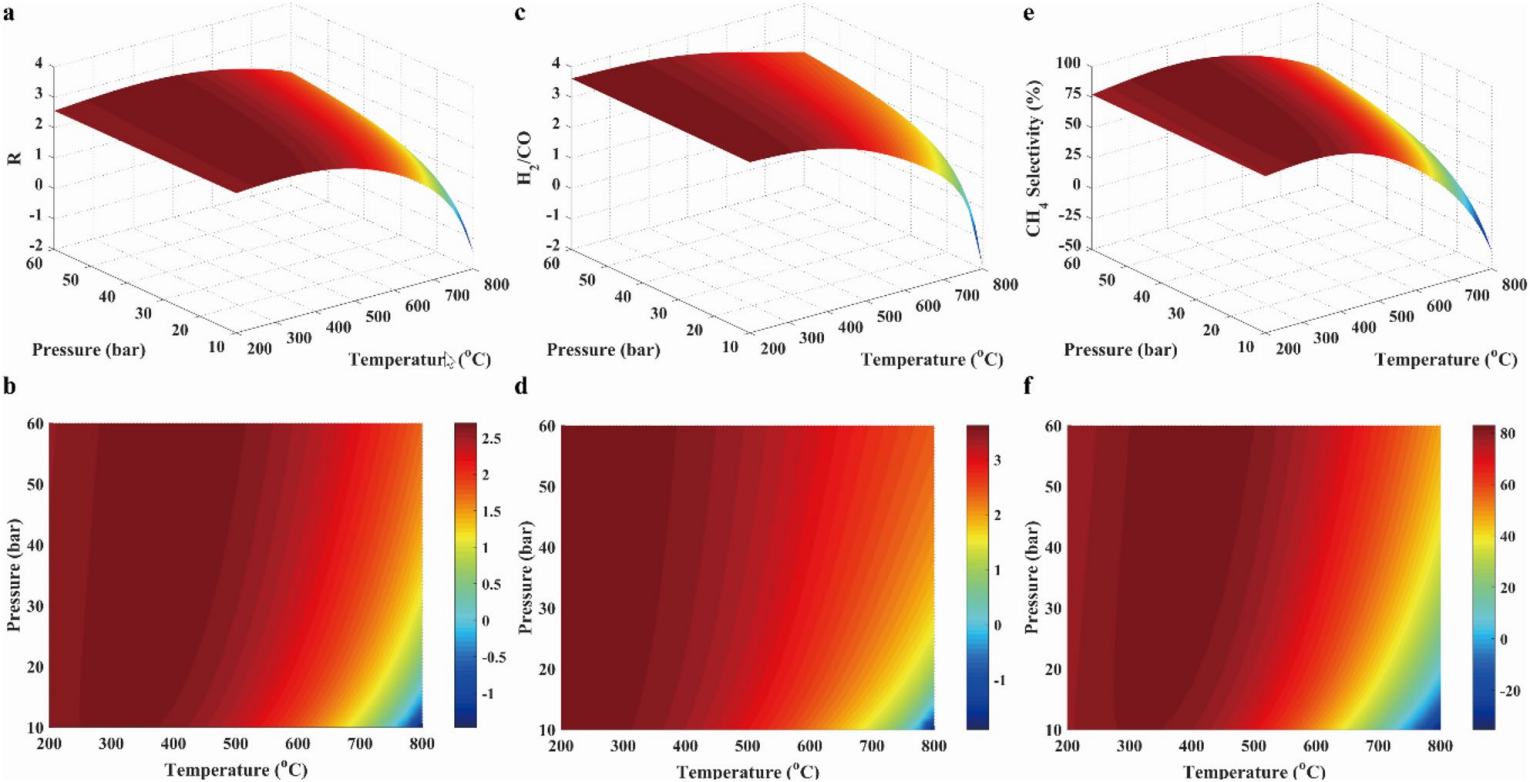
Results of *R* parameter (**a**, **b**), H_2_/CO ratio (**c**, **d**) and CH_4_ selectivity (**e**, **f**) in case 1.

To determine the suitable gas composition, the *R* parameter was evaluated. Moreover, the CH_4_ selectivity was also examined in this work. Those results were shown with 3D surfaces, which were composed by functions of temperature and pressure, as shown in Fig. 5. The* R* value was nearly equal to the 3.2 over temperature range. Besides, CH_4_ selectivity was approximated to 80% at below the temperature of 650 °C and above the pressure of 30 bar. Thus, operating conditions were thus determined.

In conclusion, a wide range of temperature and pressure can be used in methanation with COG, but the ideal condition should be controlled at the temperature below 650 °C and the high pressure. In addition, it was well known that the high pressure implied the high energy to be needed, which resulted in the high operation cost. Therefore, methanation processes were usually carried out at the mild range (15–30 bar) of pressure^31^ for economic reason.

### Methanation of COG and BOFG, case 2

By considering the high hydrogen content in COG and the high carbon content in BOFG as presented in Table 1, BOFG and COG were mixed and used to synthesize CH_4_. As discussed above, pressure was insignificant to the methanation performance, thus it was set to be 30 bar for case 2. The temperature varied from 200 to 800 °C, and BOFG/COG ratio varied within 0.0–2.0, and the similar analysis was performed as same as case 1.

**Figure 6:**
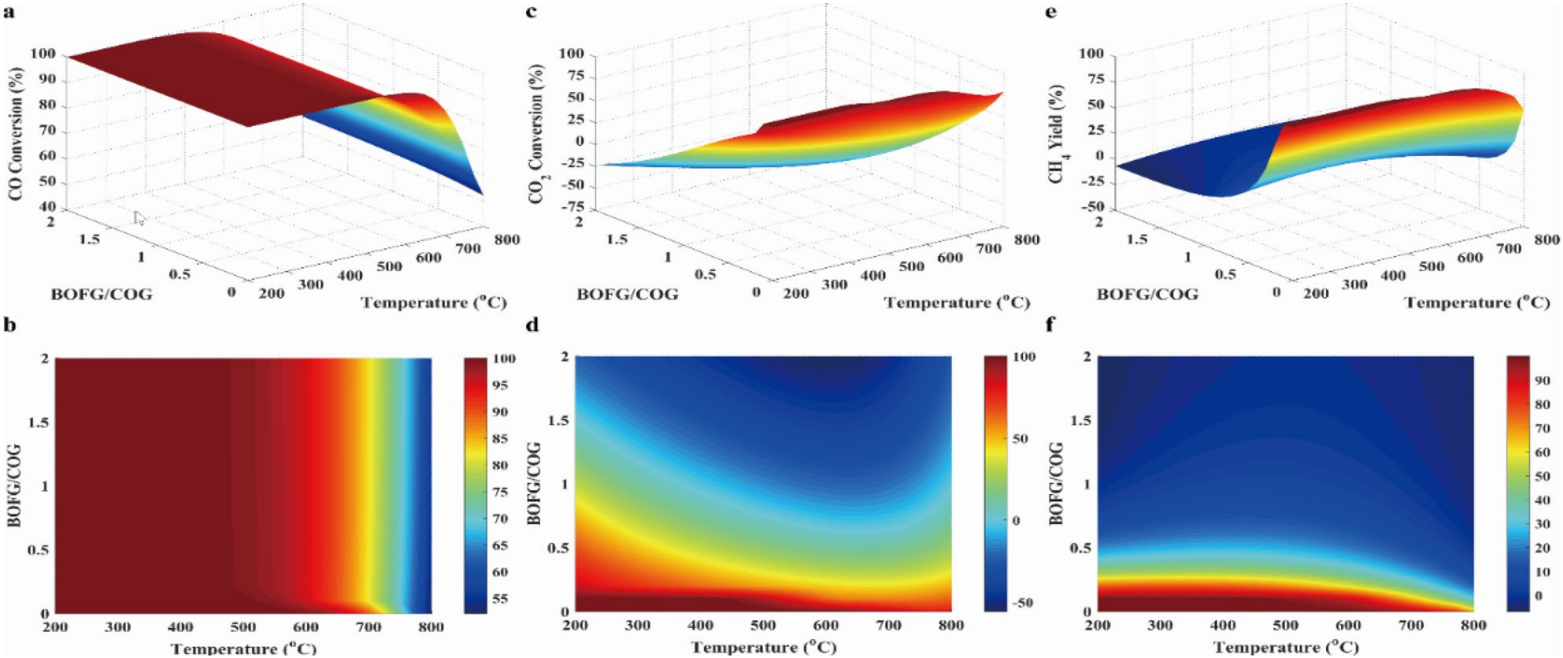
Results of the CO conversion (**a**, **b**), the CO_2_ conversion (**c**, **d**) and CH_4_ yield (**e**, **f**) in case 2.

Conversions of CO and CO_2_, and CH_4_ yield were presented in Fig. 6. As can be seen in Fig. 6, conversion and yield were favored at low temperatures since methanation was exothermic. When the BOFG/COG ratio and the temperature were less than 0.1 and 650 °C, it was found that conversion of CO_2_ and yield of CH_4_ were nearly equal to 90% and 80%, respectively. Thus, the small BOFG/BOG ratio was selected for methanation when BOFG and COG were used as the feeding gases. It was further noticed that there were negative values in CO_2_ conversion and CH_4_ yield, which could be explained by the occurrence of reactions R7 and R12.

**Figure 7:**
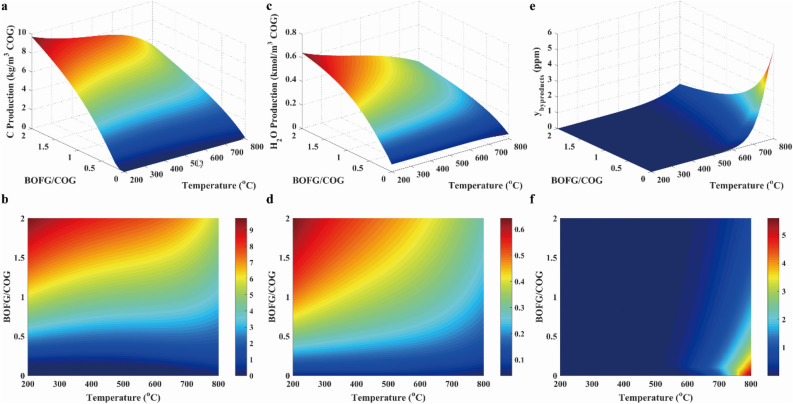
Results of carbon production (**a**, **b**), water production (**c**, **d**) and other byproducts production (**e**, **f**) in case 2.

As shown in Fig. 7a and b, carbon concentration was increased with the rising temperature because of the endothermic reaction of R12. As shown in Fig. 7c and d, H_2_O concentration was favored at the high BOFG/COG ratio, and was little changed with the increasing temperature at a low BOFG/COG ratio. As illustrated in Fig. 7e and f, the other products were ignored since their sum concentrations were smaller than 6 ppm. Therefore, the low BOFG/COG ratio was selected to reduce undesirable products.

**Figure 8:**
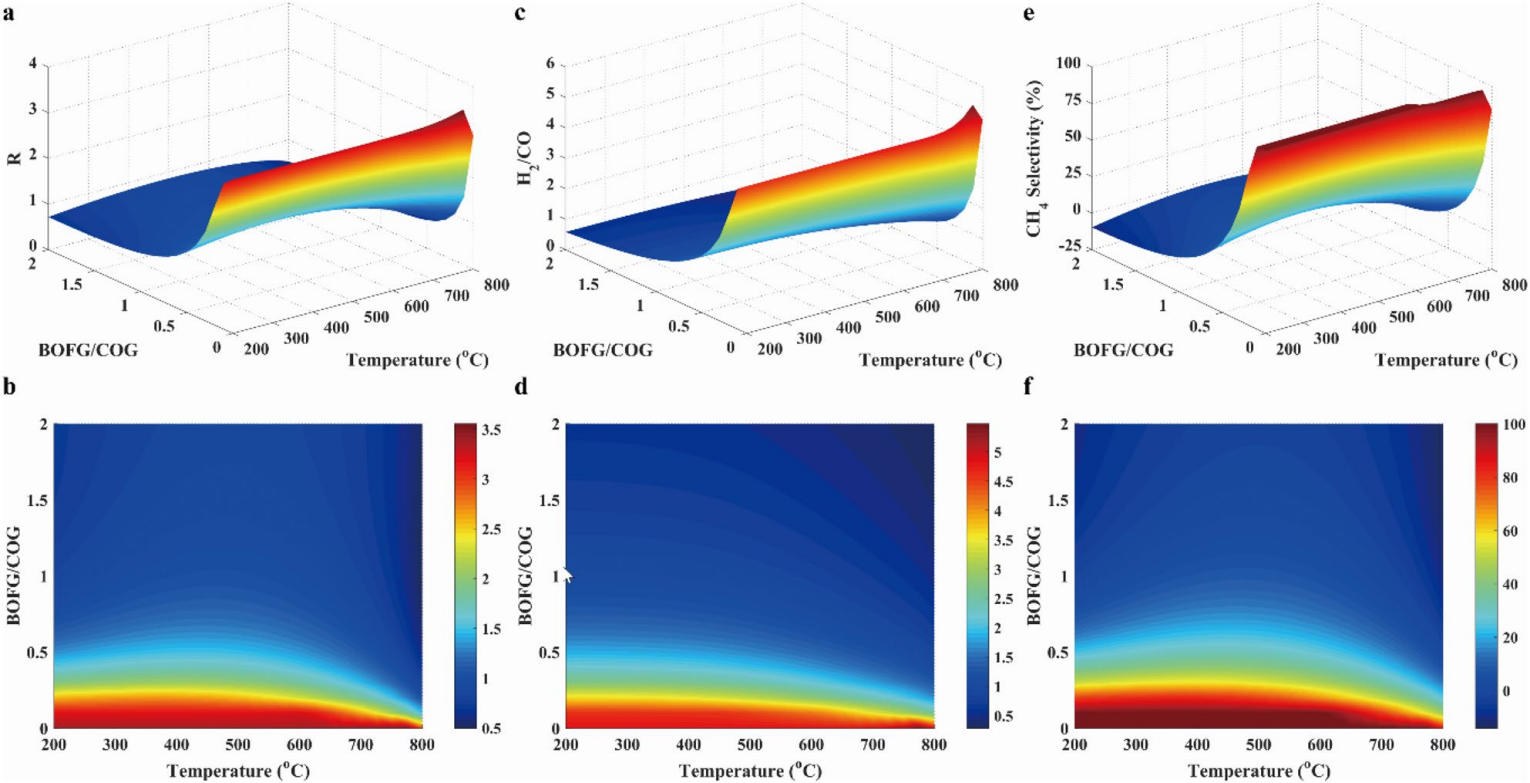
Result of R parameter (**a**, **b**), H_2_/CO ratio (**c**, **d**) and CH_4_ selectivity (**e**, **f**) in case 2.

As can be seen from Fig. 8, the *R* parameter was approximated to 3.1 over the entire temperature range when the BOFG/COG ratio was lower than 0.2. The *R* parameter was close to 3.0 when the BOFG/COG ratio was lower than 0.1 at 650 °C. In addition, CH_4_ selectivity was almost 80% when the BOFG/COG ratio was less than 0.2 at 650 °C. Thus, the temperature and the pressure of methanation conditions were less than 650 °C and 30 bar, respectively, the BOFG/COG ratio was equal to 0.1

### Methanation of COG and BFG, case 3

Since COG was rich in hydrogen and BFG was abundant with carbon, they were mixed and used as the feeding gases for methanation. As discussed above, the operating pressure was determined to be 30 bar for case 3. The varying ranges of temperature and BFG/COG ratio were identical with case 2.

**Figure 9:**
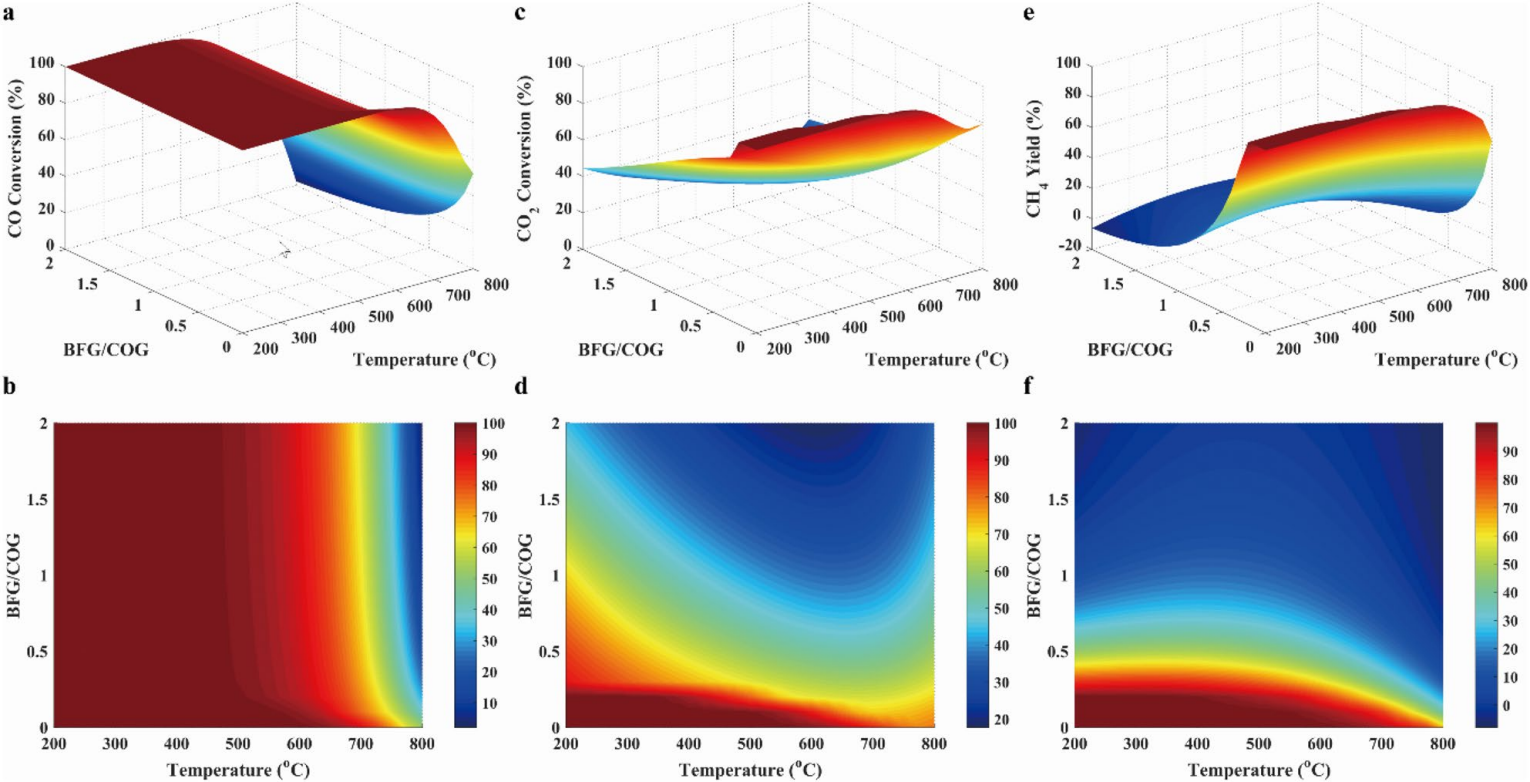
Results of CO conversion (**a**, **b**), the CO_2_ conversion (**c**, **d**) and CH_4_ yield (**e**, **f**) in case 3.

CH_4_ yield, conversions of CO and CO_2_ were provided in Fig. 9. Similar to case 2, the low temperature was favored to the conversions of CO and CO_2_, as well as CH_4_ yield. Because the methanation was exothermic. Moreover, the conversions of CO and CO_2_, and yield of CH_4_ were almost 90%, 80% and 90%, respectively, when BOFG/COG ratio was less than 0.1 with the temperature of 640 °C. Thus, the low BOFG/BOG ratio was chosen for COG methanation with BFG. It was likewise observed that there were the negative values of CH_4_ yield due to the occurrence of reaction R12. Further, CO conversion was greater than zero due to the low CO_2_ concentration in feeding gas.

**Figure 10:**
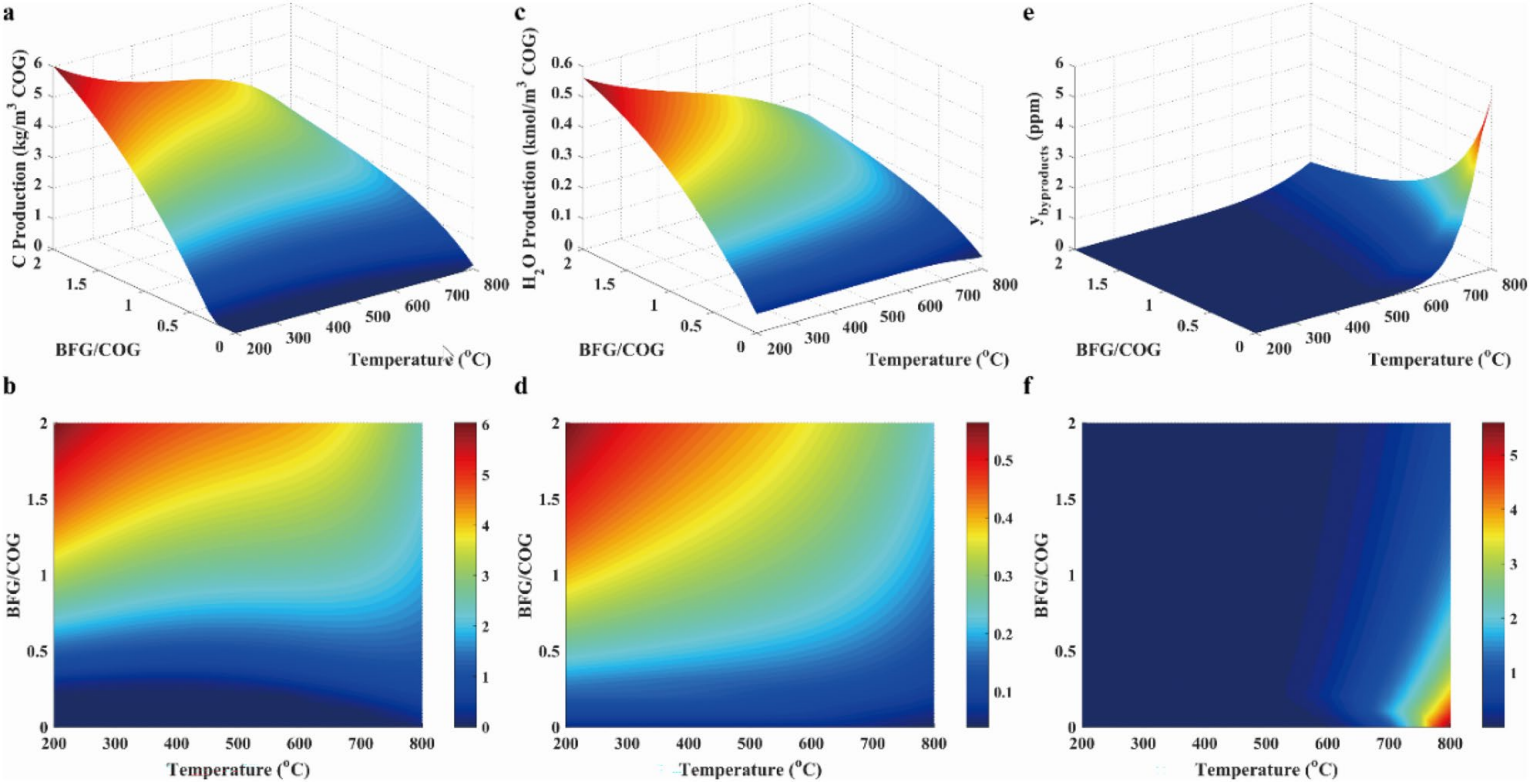
Results of carbon production (**a**, **b**), water production (**c**, **d**) and other byproducts production (**e**, **f**) in case 3.

As illustrated in Fig. 10a and b, the carbon concentration was decreased with the rising temperature because of the endothermic reaction R12. As presented in Fig. 10c and d, the H_2_O concentration was favored at the high BFG/COG ratio, and were slightly changed with the increasing temperature at low BFG/COG ratios. As given in Fig. 10e and f, the other products were ignored because of their low concentration. Therefore, the low BFG/COG ratio was selected to reduce undesirable products.

**Figure 11:**
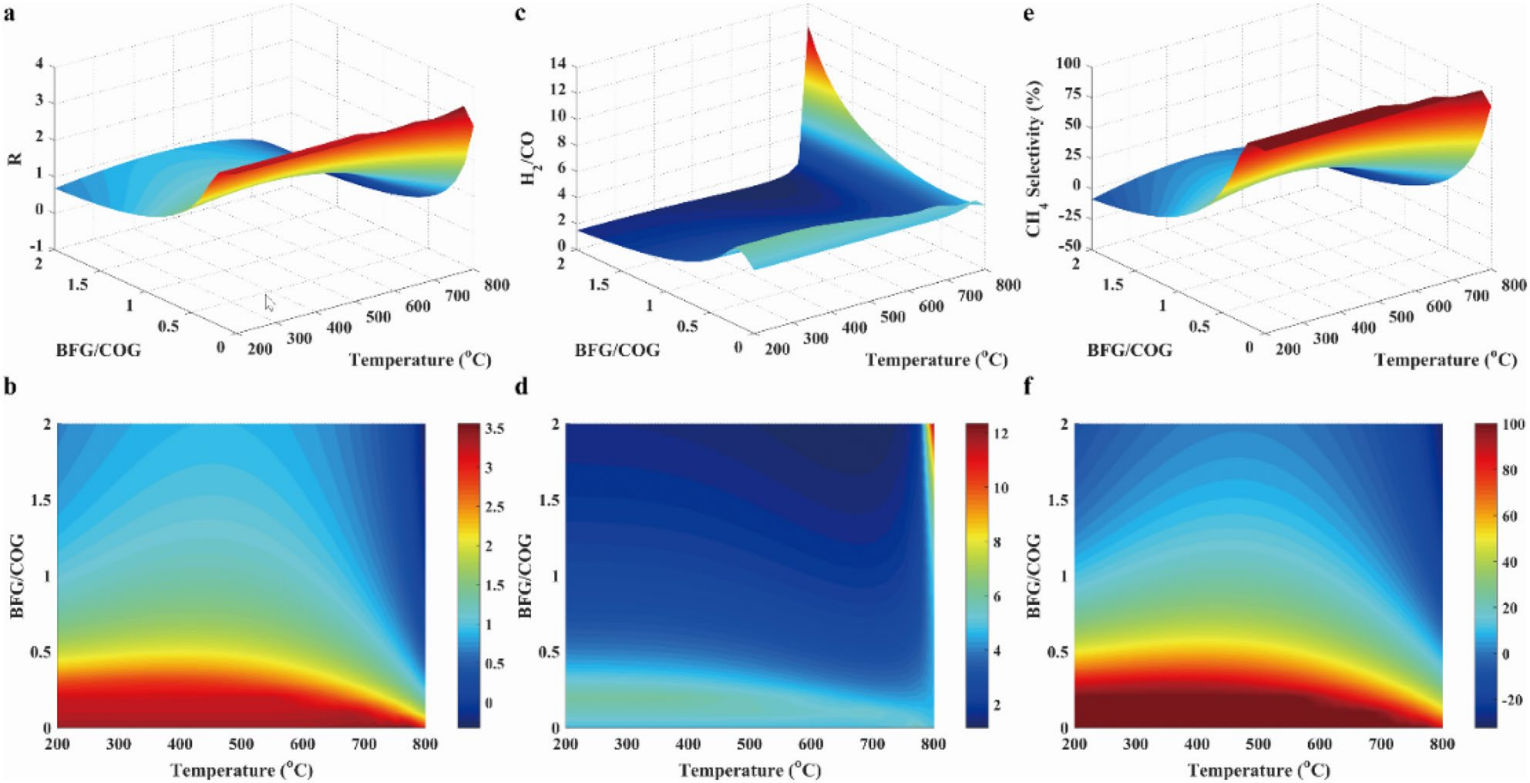
Results of R parameter (**a**, **b**), H_2_/CO ratio (**c**, **d**) and CH_4_ selectivity (**e**, **f**) in case 3.

As seen from Fig. 11, the *R* parameter was nearly equal to 3.3 over the entire temperature range. However, the *R* parameter was 0.8 at the BFG/COG of 2.0, because of the occurrence of hydrogen production from reactions R3, R4 and R11 under unsuitable conditions. Then, the *R* parameter was close to 3.0 with the BFG/COG ratio less than 0.1 at 650 °C, and the CH_4_ selectivity was approximated to 90% under the conditions. Thus, operating conditions for methanation with BFG/COG were determined at 650 °C and 30 bar with the BFG/COG ratio of 0.1.

### Methanation of COG, BFG and BOFG, case 4

**Figure 12:**
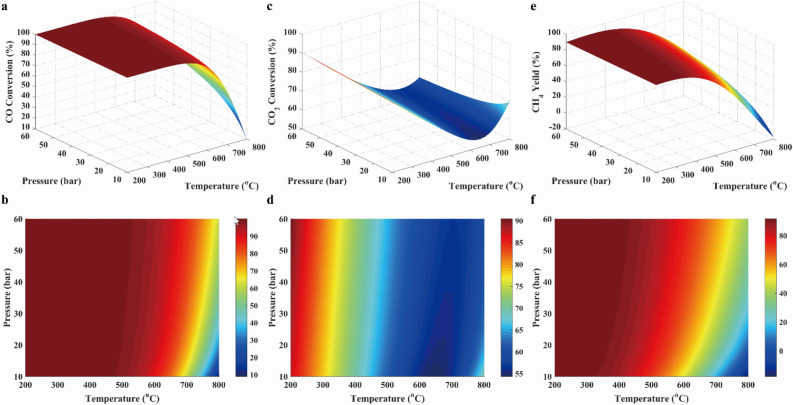
Results of the CO conversion (**a**, **b**), the CO_2_ conversion (**c**, **d**) and CH_4_ yield (**e**, **f**) in case 4.

As displayed in Table 1, the BOFG and the BFG were used to supplement the carbon source of COG methanation. Conversions of CO and CO_2_, as well as CH_4_ yield were shown in Fig. 12. As same as above cases, the operating pressure was set at 30 bar. In Fig. 12a, the conversion of CO was almost equal to 100% below 650 °C, while the conversion of CO_2_ was lower than 65% above 650 °C. Therefore, 650 °C was chosen as the methanation temperature by considering the reaction equilibrium and the reaction rate. The CH_4_ yield was close to 80% at the chosen conditions.

**Figure 13:**
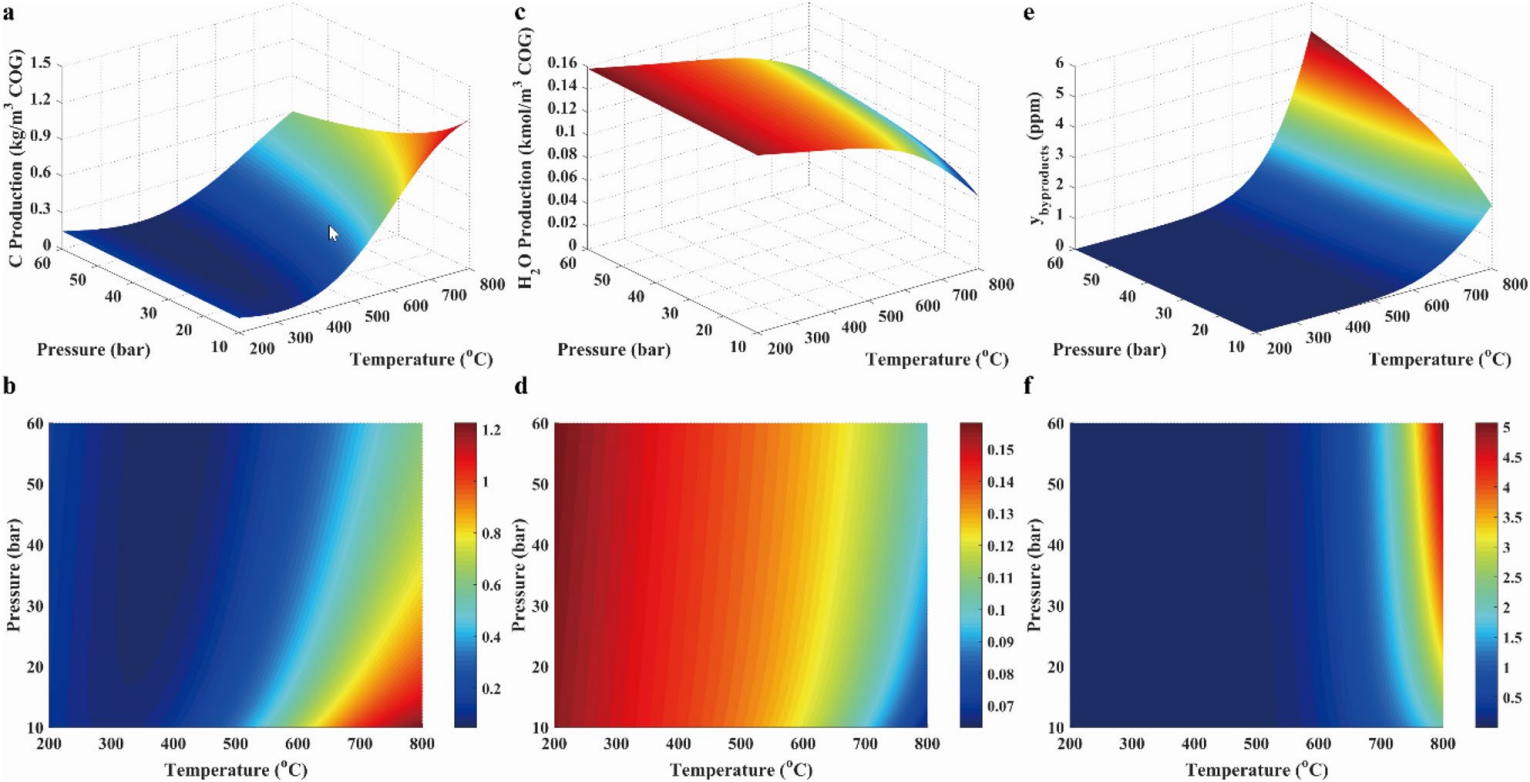
Results of carbon production (**a**, **b**), water production (**c**, **d**) and other byproducts production (**e**, **f**) in case 4.

**Figure 14:**
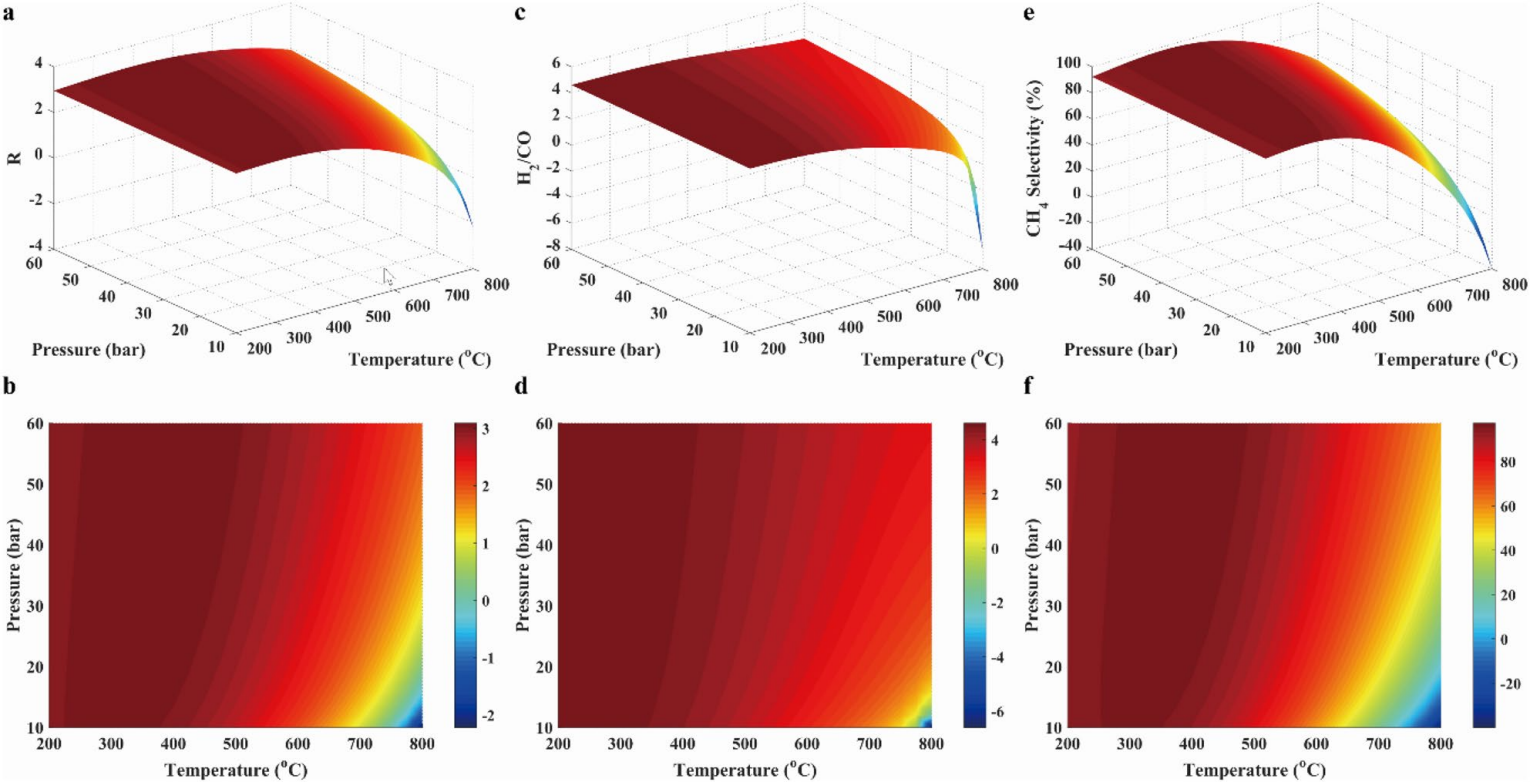
Results of *R* parameter (**a**, **b**), H_2_/CO ratio (**c**, **d**) and methane selectivity (**e**, **f**) in case 4.

As exhibited in Fig. 13a and b, the solid carbon concentration was increased with the rising temperature and the reducing pressure because the increased volume and endothermic reaction of R12 took place. As displayed in Fig. 14c and d, H_2_O concentration was increased with the decreasing temperature due to the exothermic reaction occurrences of R1 and R2. By combining Fig. 13c and d, H_2_O production was increased slightly with the rising pressure. As shown in Fig. 13e and f, other products at ppm level were ignored. Because H_2_O was benefited to inhibit the solid carbon formation reaction, the low temperature and the low pressure were chosen as operating conditions using COG, BFG and BOFG as feeding gases.

As exhibited in Fig. 14a and b, the *R* parameter was approximated to 3.1 at below 650 °C over almost the entire range of pressure. As shown in Fig. 14c and d, the low temperature was advantageous to the methane production because the high ratio of H_2_/CO was beneficial to the enhancement of methane yield. However, the low temperature resulted in the low reaction rate. Thus, there were the reasonable temperature during the methanation process of COG, BFG and BOFG. The CH_4_ selectivity was greater than 75% at below 650 °C and above 30 bar, as presented in Fig. 14e and f. Therefore, the low temperature and the high pressure were favorable for the methanation process. Considering the reaction equilibrium and the reaction rate, 30 bar and 650 °C were chosen for Case 4.

**Figure 15:**
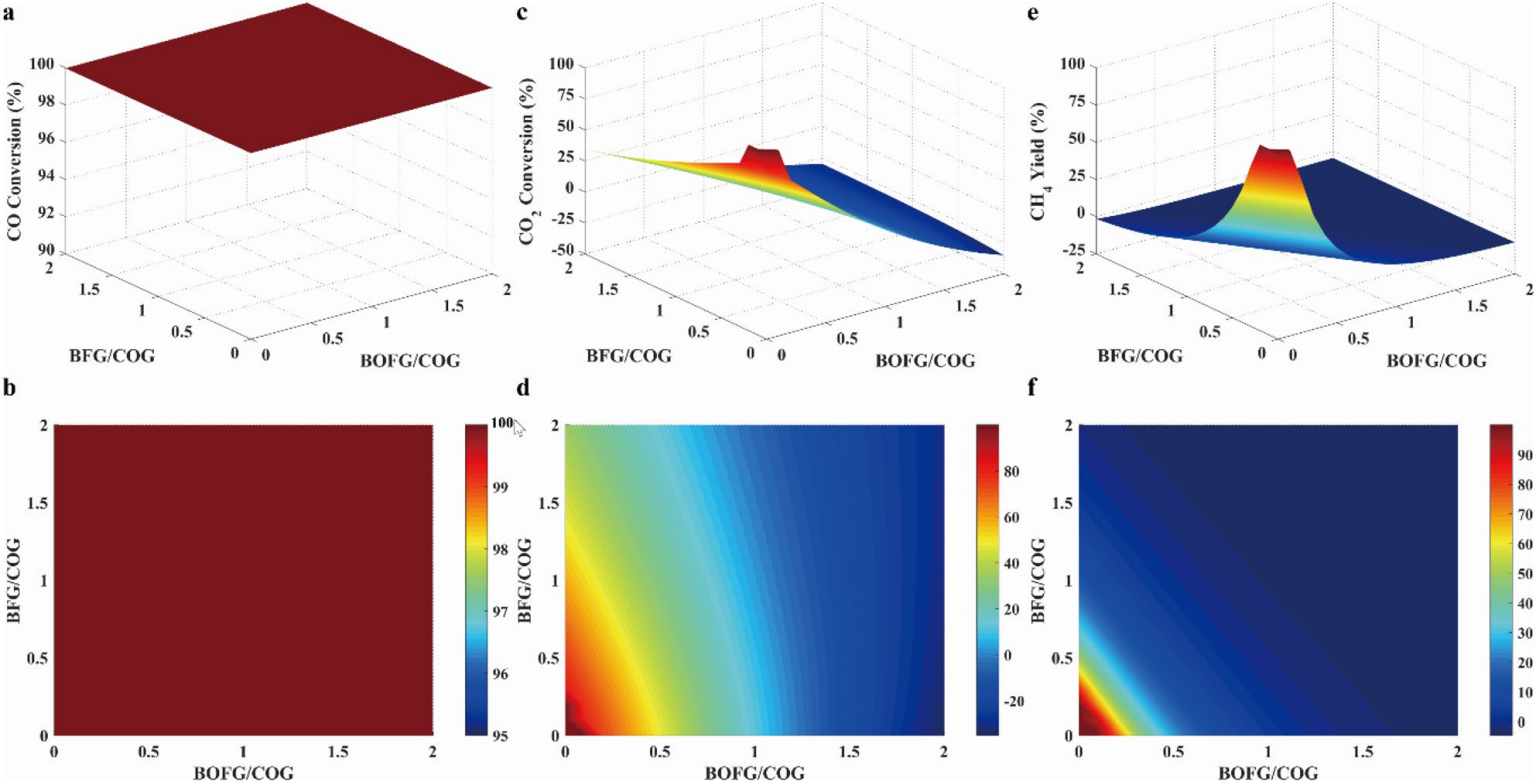
Results of the CO conversion (**a**, **b**), the CO_2_ conversion (**c**, **d**) and CH_4_ yield (**e**, **f**) at different feeding ratios.

At selected conditions (30 bar and 650 °C), the effect of BFG/COG and BOFG/COG ratios on conversion, yield, byproduct, selectivity and* R* parameter were examined as shown in Fig. 15. As shown in Fig. 15a and b, it was found that CO conversion was almost 100% under all feeding ratios, which indicated that CO methanation easily took place^31^. When the ratios of BFG/COG and BOFG/COG were both 0.1, CO_2_ conversion was mostly above 80% in Fig. 15c and d. As presented in Fig. 15e and f, it was also found that the high CH_4_ yield was favored at low ratios of BFG/COG and BOFG/COG.

**Figure 16:**
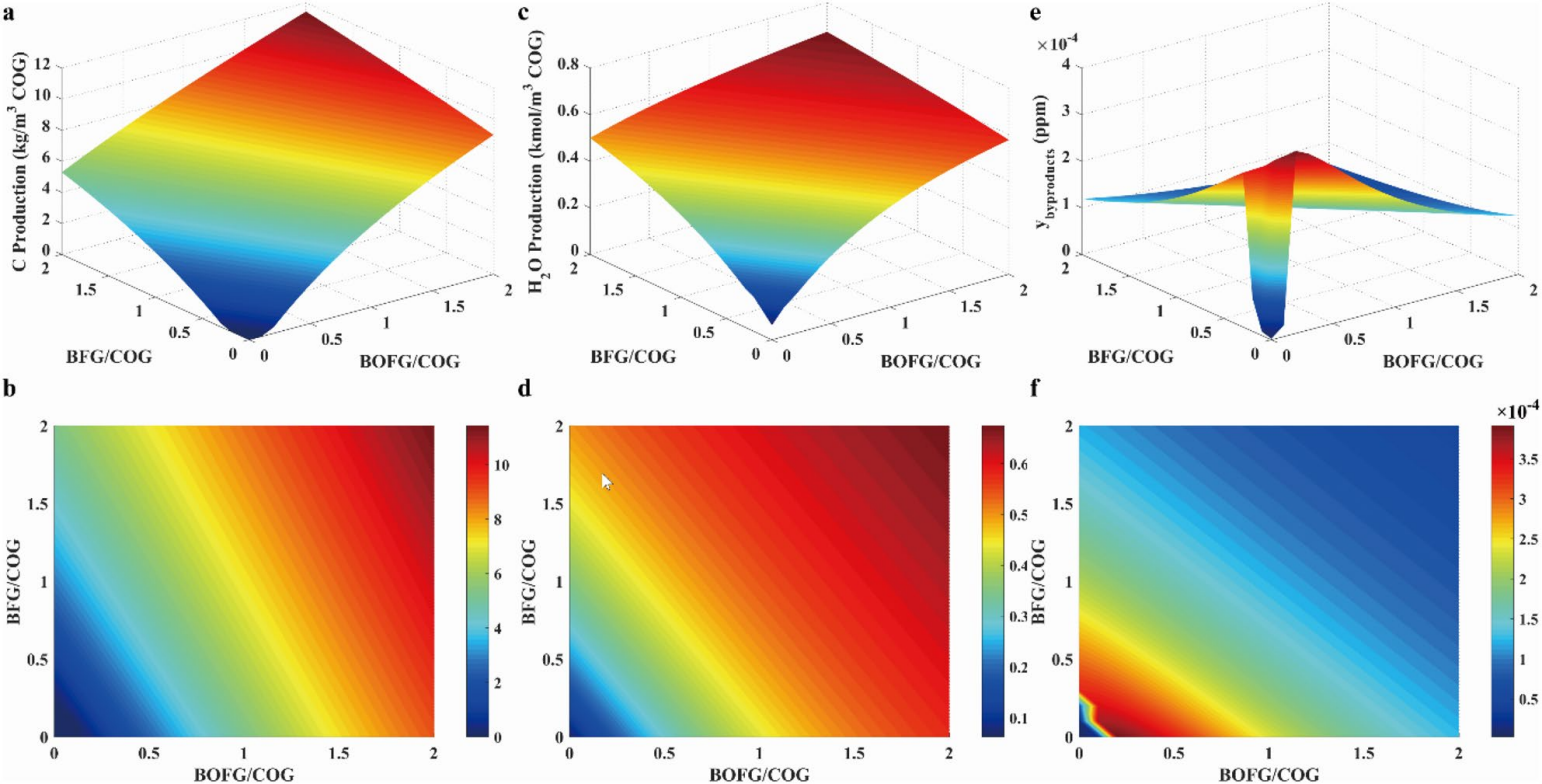
Results of carbon production (**a**, **b**), water production (**c**, **d**) and other byproducts production (**e**, **f**) at different feeding ratios.

Byproduct concentrations in feed out stream were presented in Fig. 16. It was found that byproducts could be neglected because of their low concentrations. The concentration variation of solid carbon was as same as H_2_O, as illustrated in Fig. 16a and c. Because the carbon concentration was much greater than H_2_O and carbon deposition significantly reduced catalyst activity, the ratios of BFG/COG and BOFG/COG with both values less than 0.1 were selected to reduce the solid carbon production.

**Figure 17:**
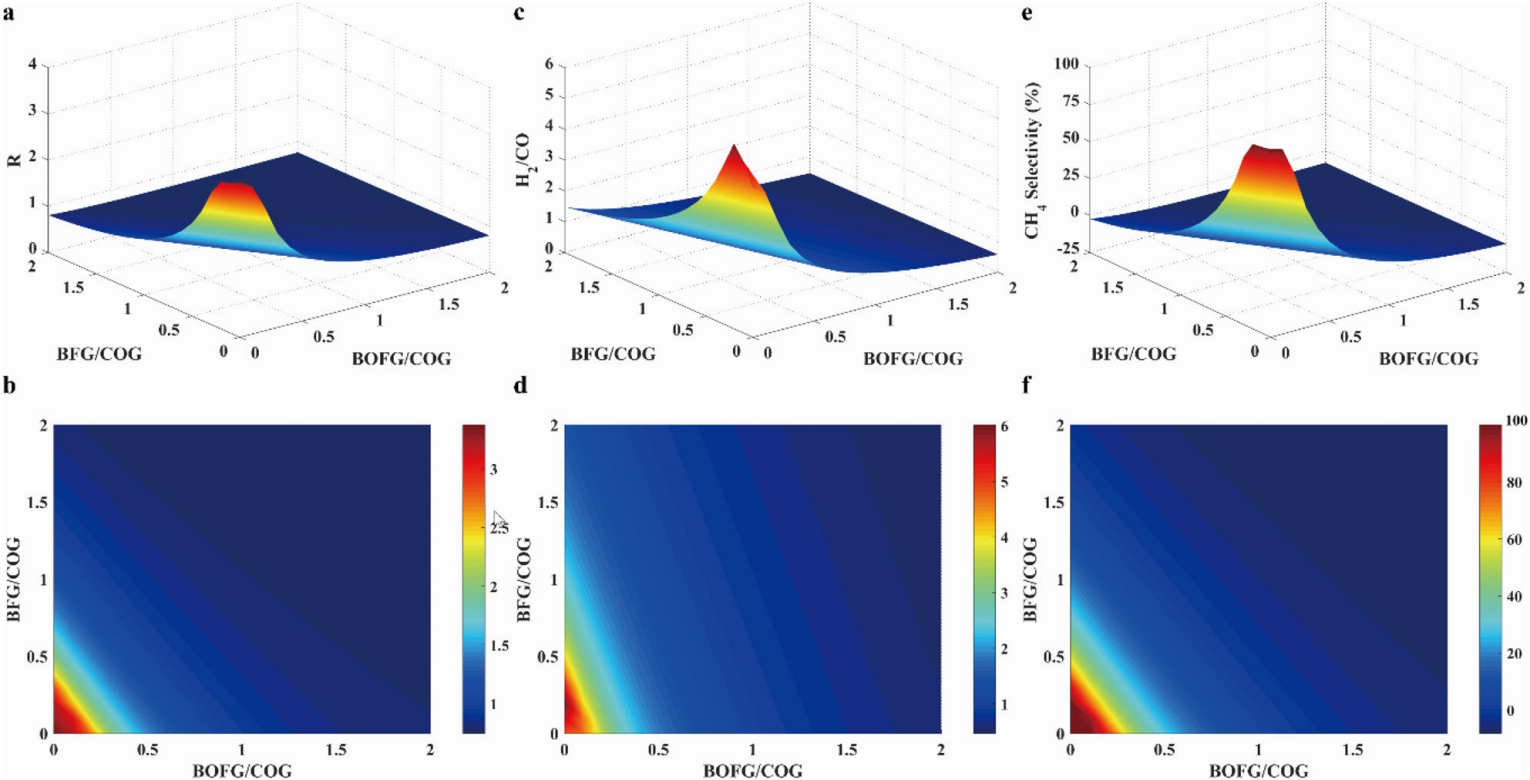
Result of R parameter (**a**, **b**), H_2_/CO ratio (**c**, **d**) and CH_4_ selectivity (**e**, **f**) at different feeding ratios.

*R* parameter, H_2_/CO ratio and CH_4_ selectivity at different feeding ratio were presented in Fig. 17. As mentioned above, it was inferred that the value of *R* parameter was equal to 3.0 under the ideal reaction condition. To satisfy the requirement of stoichiometric number, the value of R parameter need to be equal to or greater than 3.0. As shown in Fig. 17a, the value of *R* parameter was greater than 3.0 when the ratios of BFG/COG and BOFG/COG were both equal to 0.1. As presented in Fig. 17c, the H_2_/CO ratio was greater than 3.0 when the ratios of BFG/COG and BOFG/COG were both equal to 0.1. As illustrated in Fig. 17e, the CH_4_ selectivity was greater than 80% with BFG/COG and BOFG/COG at 0.1, respectively. Therefore, it was found that the ratios of BFG/COG and BOFG/COG were both equal to 0.1.

## Validation of methanation simulation

Model validation was the process, in which the modeling results were compared with the industrial or experimental or published data. If the difference between modeling results and above data was within the acceptable error margins, the proposed model can be used to present the real system behavior. Since the stoichiometric number was used to describe the molar fraction of reactant in the mixed feedstock that was consisted by the different type of gas in the united steel industry^3,4,8,32^, the stoichiometric number (*R*), the ratios of BFG/COG and BOFG/COG, the operating temperature and the pressure were selected to validate the simulated results with industrial data and published literature^3,33^, as shown in Table 4. As illustrated in Table 4, it was found that the good agreement was observed between the industrial or published data^3,33^ and data obtained from the proposed model. It was seen that the maximum relative error was 16.7% for the pressure, which could be ignored because of its insignificant effect on methanation. For practice, the temperature of methanation process was slightly lower than the thermodynamic prediction. Because the low temperature was advantage to the enhancement of methane conversion.

**Table 4:** Validation of the simulating methanation results.

Source	Temperature (°C)	Pressure (bar)	R	BFG/COG	BOFG/COG	Temp relative error (%)	Press relative error (%)	R relative error (%)
Literature^3^	700	25	3.0	**–**	**–**	7.1	16.6	0
Literature^33^	680	25	3.1	**–**	**–**	4.4	16.6	3.3
Northwest Res. Inst. Chem. Ind	650	31	3.0	**–**	**–**	0	3.2	0
Shanxi Juyuan Coal Chem. CO. LTD	650	25	3.0	**–**	**–**	0	16.6	0
Southwest Res. Des. Inst. Chem. Ind	650	32	3.0	10%	**–**	0	6.3	0
Chengdu Tongchuang Weiye New Energ. Tech. CO. LTD	630	30	3.0	**–**	10%	3.1	0	0
Simulated results	650	30	3.0	10%	10%	**–**	**–**	**–**

## Conclusions

The optimization mathematic model based on Gibbs free energy minimization was established to predict the thermodynamic feasibility of coke oven gas (COG) methanation, which was combined without and with the blast furnace gas (BFG) and the basic oxygen furnace gas (BOFG). To solve the proposed model, the comfortable method was proposed, which was implemented by using the Gibbs module in Aspen Plus software. Further, effects of operation parameters on methanation performance were revealed to identify the optimized operating conditions of COG methanation. To improve the performance of COG methanation, it was found from estimated results that the optimal reaction temperature, the optimized operating pressure and the optimal stoichiometric number were 650 °C, 30 bar and 3.0, respectively. Moreover, it was discovered that 10 mol % of BFG or BOFG could be mixed into COG to obtain the optimized methane yield. In addition, it was testified that there were the good agreements between calculated results and industrial and published data, which indicated that the methanation simulations of COG combined with and without BFG and BOFG were valid. Also, it was found that BFG and BOFG were used as carbon resource to adjust the H_2_/CO ratio for the suitable stoichiometric number, which improved the utilization rate of material and energy in steel plant by-product gases. Specifically, the CCUS technology was used to supply the carbon resource in the COG methanation technology, which was a very valuable method in viewpoint of economy and environment.

### Supplementary Information

Supplementary Information 1.

Supplementary Information 2.

Supplementary Information 3.

Supplementary Information 4.

## Data Availability

The data sets generated and analyzed in this study will not be made public for privacy reasons, but data may be obtained from the corresponding author upon reasonable request.
